# Utilization of HIV and Tuberculosis Services by Health Care Workers in Uganda: Implications for Occupational Health Policies and Implementation

**DOI:** 10.1371/journal.pone.0046069

**Published:** 2012-10-11

**Authors:** Esther Buregyeya, Fred Nuwaha, Rhoda K. Wanyenze, Ellen M. H. Mitchell, Bart Criel, Suzanne Verver, Simon Kasasa, Robert Colebunders

**Affiliations:** 1 Makerere University School of Public Health, Kampala, Uganda; 2 KNCV Tuberculosis Foundation, The Hague, The Netherlands; 3 Institute of Tropical Medicine, Antwerp, Belgium; 4 University of Antwerp, Antwerp, Belgium; University of Ottawa, Canada

## Abstract

**Background:**

Access to HIV testing and subsequent care among health care workers (HCWs) form a critical component of TB infection control measures for HCWs. Challenges to and gaps in access to HIV services among HCWs may thus compromise TB infection control. This study assessed HCWs HIV and TB screening uptake and explored their preferences for provision of HIV and TB care.

**Methods:**

A cross-sectional mixed-methods study involving 499 HCWs and 8 focus group discussions was conducted in Mukono and Wakiso districts in Uganda between October 2010 and February 2011.

**Results:**

Overall, 5% of the HCWs reported a history of TB in the past five years. None reported routine screening for TB disease or infection, although 89% were willing to participate in a TB screening program, 77% at the workplace. By contrast, 95% had previously tested for HIV; 34% outside their workplace, and 27% self-tested. Nearly half (45%) would prefer to receive HIV care outside their workplace. Hypothetical willingness to disclose HIV positive status to supervisors was moderate (63%) compared to willingness to disclose to sexual partners (94%). Older workers were more willing to disclose to a supervisor (adjusted prevalence ratio [APR] = 1.51, CI = 1.16–1.95). Being female (APR = 0.78, CI = 0.68–0.91), and working in the private sector (APR = 0.81, CI = 0.65–1.00) were independent predictors of unwillingness to disclose a positive HIV status to a supervisor. HCWs preferred having integrated occupational services, versus stand-alone HIV care.

**Conclusions:**

Discomfort with disclosure of HIV status to supervisors suggests that universal TB infection control measures that benefit all HCWs are more feasible than distinctions by HIVstatus, particularly for women, private sector, and younger HCWs. However, interventions to reduce stigma and ensuring confidentiality are also essential to ensure uptake of comprehensive HIV care including Isoniazid Preventive Therapy among HCWs.

## Introduction

High rates of tuberculosis (TB) disease among health care workers (HCWs) have been reported in African countries, with HIV-infected HCWs at extremely high risk [1], [2], [3]. The World Health Organization (WHO) and the International Labour Organization (ILO) recommend surveillance of HCWs for TB disease as well as encouraging them to know their HIV status [4], [5]. If diagnosed with HIV, HCWs should be offered a package of TB prevention and care that includes regular screening, access to antiretroviral therapy (ART) and Isoniazid Preventive Therapy (IPT). In addition, HIV positive HCWs should be given a choice to opt out of working in areas where they are exposed to infectious TB especially multidrug resistant TB (MDR-TB) and extensively drug-resistant TB (XDR-TB), and offered a position from where exposure to untreated TB is low. HIV prevalence in Uganda has stagnated at 6.7%[6]. However the HIV prevalence among HCWs in Uganda is unknown. Studies conducted elsewhere have reported that HCWs prevalence mirrors that in the community [7], [8]. Previous studies have reported challenges HCWs face to access general or workplace HIV services [9], [10]. Such challenges include: stigma, a feeling of professional failure, shame, and fear to be known as HIV infected by colleagues and patients [9], [10]Uganda is ranked 18^th^ among the 22 TB high burden countries in the world [11]. About 54% of TB cases in Uganda are co-infected with HIV and about 30% of the HIV-related deaths are attributed to TB [12]. Despite this high TB burden, not many studies have been conducted on TB among HCWs in Uganda. In a study by Kayanja [13], 57% of the HCWs had a tuberculin skin test results ≥10 mm. Recently the Ministry of Health of Uganda and the Tuberculosis Control Assistance Programme (TBCAP) started to emphasise TB infection control (IC) interventions; other than training of HCWs, so far very few other interventions have been implemented.

HIV counselling and testing (HCT) acts as an entry point into TB IC, since the package of TB IC depends on the HIV status of the HCWs, in addition to the general administrative, ventilation and respiratory protective equipment [4]. For the HIV infected HCWs, the TB IC package consists of regular screening for TB (disease and infection), access to HIV care and ART as well as IPT. Additionally, HIV infected HCWs working in areas with high TB risk should ideally have the option to be relocated to a low-risk area in the health facility. However, this may require disclosure of their HIV positive status to their supervisors as these supervisors/managers generally make decisions about the allocation of duties and sites. The need for HIV testing and disclosure among HCWs, makes implementation of TB IC more complex, because of confidentiality considerations and the stigma that may be associated with HIV testing and access to HIV care and treatment. The challenges encountered by HCWs in this trajectory, and their preferences in terms of how HIV and TB services should be delivered are not well documented. The aims of this study were to document HCWs utilisation of HIV and TB screening services (the level of utilisation, where they tested from, the circumstances around the testing, willingness to disclose HIV positive status to their supervisor). In addition, we explored their preferences for accessing HIV and TB services.

## Methods

### Ethics Statement

The study was approved by the Makerere University School of Public Health Higher Degrees Research and Ethics Committee and the Uganda National Council for Science and Technology. Informed written and verbal consent was obtained for the survey and FGDs, respectively, from participants at the time of data collection.

### Study design, setting and population

We conducted a cross-sectional study among HCWs in health facilities in the districts of Mukono and Wakiso in central Uganda, from October 2010 to February 2011. These two districts surrounding Kampala, the capital city are partly semi-urban but predominantly rural [14]. The HIV prevalence among the general population is estimated at 6.5% in Mukono and 8.5% in Wakiso [15]. Both districts have a low TB case-detection rate: 46% in Mukono and 38% in Wakiso (Ministry of Health, unpublished report, 2008) compared to the WHO TB global target of 70% [16]. In the two districts, training in TB IC was conducted 1–2 years prior to this survey. The training was conducted by the Uganda Ministry of Health and TBCAP in an effort to implement TB IC. The objectives of the training were to teach HCWs: 1) to conduct a TB IC assessment in a health facility and 2) to develop and implement a TB IC plan [17]. Facilities were asked to send up to two people (usually a TB focal person and a laboratory technician) to attend the training. The trained individuals were expected to train other HCWs, upon return to their health facilities. During this training HCWs were encouraged to know their HIV status, but HIV testing, care and treatment were not offered as part of the intervention.

Health care in Uganda is provided by both public and private sector (private-not-for-profit - PNFP and private for profit). Uganda has a decentralised public health care system. All PNFP and public health facilities from sub-county health facility (HC III) to hospital level (excluding those located on islands because of accessibility challenges) in both districts were included in the study. All study health facilities had HCT services, but none was providing IPT.

### Data collection

Both quantitative and qualitative data collection methods were used because of increasing recognition that a mixed-methods approach yields richer findings and improves interpretation of results [18], [19]. The qualitative methods comprised Focus Group Discussions (FGDs) which were conducted concurrently with the questionnaire survey. Each method asked distinct, but related questions

### Quantitative survey

All health facilities in the two districts, both public and private-not-for-profit (PNFPs) were included in the study. To develop a sampling frame, lists of all health facilities within each district and the list of staff at each public facility were obtained from the district health offices. For PNFP facilities, the lists of staff were obtained on-site. In total, the study was undertaken in 51 health facilities. Based on the total number of HCWs in a district and at each facility, the sample for each facility was determined in order to guarantee an equal probability of selection. At the facility level HCWs were stratified by cadre in order to obtain a proportional representation of each staff category (doctors, clinical officers, nursing, midwifery, nursing aid, laboratory and radiographers). Using simple random sampling, respondents were selected from each category. In lower level facilities, (some county based health centres/HC IVs and all sub-county-based health centres/HCIII) where virtually all staff were in the nursing cadre, simple random sampling was used to select the respondents. Our calculated sample size was 551 HCWs, assuming that 80% of HCWs had ever tested for HIV [20], with a 95% confidence interval, design effect of 2 and a 10% adjustment for non-response. Data were collected using an anonymous self-administered structured questionnaire. Data were collected on socio-demographic characteristics of HCWs, history of HIV and TB screening (disease and infection), where they tested and their willingness to disclose an HIV positive status. In addition, HCWs were asked whether they had suffered from TB in the last five years, and their preferences in accessing HIV and TB services.

### Qualitative data

FGDs with the use of an interview guide, explored health workers' perspectives on utilisation of HIV services, access to confidential HIV services in their facilities, disclosure of HIV status, and their opinions on preferences for HIV and TB services for them. FGDs were only conducted in large health facilities which had sufficient number of HCWs to constitute an FGD. Eight FGDs were conducted with HCWs (different from those who participated in the survey). Seven FGDs were conducted with female nurses (working in outpatient departments, HIV clinics, medical wards and TB clinics) and one with males (a mix of laboratory personnel, nurses and clinical officers). Only one male FGD was conducted because it was difficult to get an adequate number of male HCWs at any one facility. Conducting FGDs by gender was necessary to create a conducive environment for participants to express themselves freely. The FGD participants were purposefully recruited with the help of the facility managers. All the discussions lasted 1–2 hours. The FGDs were carried out by two trained research assistants (one male and another female) and the lead author. During the FGDs, one research assistant facilitated the discussion, while the other did the note taking, in addition to audio recording. These methods have been previously described [21].

### Quality control

Study tools (questionnaire and interview guide) were pretested and revised accordingly. Research assistants with a degree and vast experience in research were trained on the objectives of the study and how to administer the tools. Although the quantitative questionnaires were self-administered, questionnaires were completed by respondents at the same time in one room. Respondents sat far from each other, in order to maintain confidentiality and avoid discussing responses. A research assistant, with support from the lead author, read through each question and provided clarifications in order to minimise skipping of questions. Quantitative data was field edited before data entry into the computer.

### Data management and analysis

Quantitative data were double entered in Epi-Info Version 3.2.2 software and cleaned before being exported to STATA version 10 for analysis. Respondents were further categorized into clinical and non-clinical cadres. Clinical cadres included doctors, clinical officers, pharmacist, midwives, registered and certified nurses, while the rest were classified as non-clinical cadres. The time interval between HIV testing and date of data collection was summarized by median with an interquartile range (IQR), because it was not normally distributed. Proportions for categorical variables were calculated. Frequencies for categorical variables were calculated with different denominators, as not all respondents answered all questions. Bivariate analysis using Chi-square test and prevalence ratios (using generalized linear model with a Poisson link) were performed. Bivariate and multivariable analysis was used to explore the factors associated with willingness to disclose HIV positive status to their supervisors and whether they would prefer to receive HIV care and treatment at their workplace. An association was considered significant at P<0.05 and 95% confidence intervals (CI). Variables that had a p-value<0.2 at bivariate level were included in the multivariable analysis model with backward elimination to find independent factors that were associated with willingness to disclose HIV positive status and preference to receive HIV care and treatment at their workplace. For qualitative data, transcriptions from the audio recordings formed the empirical basis for the content analysis [22]. Transcripts were first read several times to get an overall picture and then recurring themes were identified [23]. Quotations that epitomised central themes were identified. Since each method asked different, but related questions, we treated the data from the two methods as complementary [18]. We presented results together where appropriate.

## Results

### General Characteristics

Overall, 543 out of 551 HCWs (98.5%) completed the questionnaire. However, 499 HCWs (92%) responded to all the required questions. Analysis was done based on 499 completed questionnaires. The majority of the respondents (72%) were females. The mean age of the respondents was 35.7 (10 SD) years and median of 34 years (IQR 28 to 43). Among the clinical cadres, the majority were nurses (51%), Table 1. The majority of the respondents (81%) belonged to the clinical cadres and this comprised of doctors (4%, 15/406), clinical officers (18%, 74/406), registered nurses (20%, 80/406), enrolled nurses (26%, 106/406), midwives (16%, 66/406) and the rest laboratory staff. Among the non-clinical cadres, 77% (72/93) were nursing aids and the rest records assistants.

**Table 1:** Socio-demographic characteristics of health care workers from Mukono and Wakiso districts in Uganda.

Variable	n (%)
**District**	
Mukono	242/499(48.5)
Wakiso	257/499(51.5)
**Facility level**	
Hospital	221/499 (44.3)
HCIV	114/499 (22.9)
HC III	164/499 (32.9)
**Facility ownership**	
Government	331/499(66.3)
PNFP	168/499 (33.7)
**Sex**	
Male	140/499 (28.1)
Female	359/499 (71.9)
**Age**	
15–24	64/499 (12.8)
25–34	180/499 (36.1)
35–44	123/499 (24.7)
44 and above	132/499 (26.4)
**Cadre**	
Clinical	406/499 (81.4)
Non-clinical	102/499 (18.6)
**Department**	
Outpatient*	427/499(85.6)
Medical ward	72/499(14.4)

HC = Health Centre, PNFP = Private-Not-For-Profit.

* *included the general outpatient department, HIV clinic, laboratory, pharmacy, records, and maternal child health services*.

### Utilisation of HCT among health care workers

Ninety five per cent of the respondents reported having ever tested for HIV, (Table 2). The median time interval between HIV testing and date of data collection was 7 months (IQR 4 to 13 months). One third of the respondents reported having tested outside their workplace. For those who tested from their places of work, more than a quarter (84/315) reported testing themselves. Reasons for testing included: wanting to know their status 365/474 (77%), for post exposure prophylaxis 32/474 (7%), marriage 30/474 (6%), availability of free testing services 48/474 (10%), planning to get a child 38/474 (8%) and for fear of getting TB 3/474 (0.6%); some respondents reported more than one reason. There was no association between ‘ever tested for HIV’ and sex, age, cadre, facility level and facility ownership.

**Table 2:** Utilisation of HIV counselling and testing services and willingness to disclose HIV positive status among health care workers in Mukono and Wakiso districts in Uganda.

Question	n	%
1. Ever tested for HIV?		
Yes	474/499	95.0
No	25/499	5.0
2. Testing venue		
Workplace	315/474	66.5
Away*	159/474	33.5
3. If the test was done at the workplace, who carried out the test?		
Myself	84/315	26.7
Lab & other colleagues	231/315	73.3
4. If the test was performed outside the place of work, where was the test done?		
Another facility	137/159	86.2
Home	22/159	13.8
5. If you were found to be HIV positive, would you tell your colleagues?		
Yes	257/499	51.5
No	242/499	48.5
6. Would you be willing to disclose your HIV positive status to your supervisor		
Willing	312/499	62.5
Not willing	187/499	37.5
7. If you had HIV, would you prefer to be treated at the facility where you work		
Yes	274/499	54.9
No	225/499	45.1

According to the FGDs, there was a general feeling that testing at one's workplace was difficult and not common. HCWs who test were believed do it outside their facility where they are not known.

“*That thing (HIV testing) is secretly done by the health workers; some test from here and others from somewhere else due to fear that they will be stigmatized. We don't test from here; everyone goes somewhere else*."- FGD females

There was a feeling that those who test from their workplaces are usually confident that they are HIV negative. Those who are not sure of their status but test at their workplace were believed to either do self testing, utilise HIV outreach services or disguise their blood samples as belonging to patients.

‘*Those who test will just say, “I have a sample from a patient that I want you to test for HIV. I came into contact with this patient's blood. I want to know his/her status*."’-FGD males.

“*Some of them come. We counsel them, but when it comes to testing, they say, ah-ah, sister I think I will come next time. There's that fear which is still within us*."-FGD females.

### Willingness to disclose HIV positive status among health workers

Almost all, respondents (94%, 468/499) reported that if they were HIV infected, they would disclose to their partners. However, willingness to disclose to colleagues and supervisors was less common, with just a half (257/499) and 63% (312/499), respectively (Table 2). Females and HCWs from PNFP compared to government facilities were less willing to disclose to their supervisors (Table 3). In addition, older HCWs were more likely to report willingness to disclose to their supervisors. In a multivariable model, older age (adjusted prevalence ratio [APR] = 1.51; CI = 1.16–1.95), was positively associated with reported willingness to disclose to a supervisor unlike being female APR = 0.78; CI = 0.68–0.91, and HCWs from PNFPs, APR = 0.81; CI = 0.65–1.00, (Table 3). Disclosure of a positive HIV status to colleagues was anticipated to be a thorny issue. The majority of the FGDs reported that disclosure of HIV status was difficult, though some felt that it would be good to be open about their status. HCWs recognized that disclosure to supervisors was beneficial in terms of getting support such as reduced workload and providing psychological relief.

**Table 3:** Bivariate and multivariable analysis of willingness to disclose an HIV positive status to a supervisor/manager among health care workers in Wakiso and Mukono in Uganda.

Characteristics	Willing to disclose (%)	Crude PR (95% CI)	Adjusted PR (95% CI)	P-value
**Age (years)**				
15–24	30/64(46.9)	1	1	
25–34	105/180(58.3)	1.24 (0.97–1.59)	1.22(0.96–1.57)	0.09
35–44	82/123 (66.7)	1.42(1.07–1.87)	1.37(1.04–1.78)	0.02
>45	95/132(72.0)	1.54(1.18–2.00)	1.51(1.16–1.95)	<0.01
**Sex**				
male	101/140 (72.1)	1	1	
female	211/359(58.8)	0.81(0.69–0.95)	0.78(0.68–0.91)	<0.01
**Ownership**				
Government	223/331(67.4)	1	1	
PNFP	89/168 (53.0)	0.78(0.63–0.98)	0.81(0.65–1.00)	0.05
**Facility level**				
Hospital	127/221 (57.5)	1	-	-
HCIV	73/114 (64.0)	1.11(0.89–1.38)		
HCIII	112/164 (68.3)	1.19(0.95–1.49)		
**District**				
Wakiso	160/257 (62.3)	1	-	-
Mukono	152/242 (62.8)	1.00(0.83–1.22)		
**Cadre**				
Non-Clinical	60/93(64.5)	1	-	-
Clinical	252/406 (62.1)	0.96(0.82–1.13)		

CI: Confidence Interval, HC = Health Centre, PNFP = Private-Not-For-Profit, PR = Prevalence Ratio.

HCWs were afraid to disclose their HIV positive status for fear of gossip, losing social status, feeling out of place in society and being isolated by colleagues, stigmatized and losing their jobs. It was reported that the association of HIV with immoral sexual behaviours made it difficult for one to declare that they are HIV infected.

“…*Although one could have got it [HIV] through a prick, no one will say that she got a prick as she was putting up a drip. They will say, ‘she messed up’*." - FGD females

Other HCWs reported that refusing to disclose is because of feeling guilty and embarrassed, since as HCWs, they should have prevented it.

On the other hand, some HCWs felt that disclosure was not necessary, since as health workers, they knew the necessary steps to take in terms of treatment and adherence to treatment. Though disclosing to their colleagues was described as optional and most times not necessary, they felt that disclosing to their supervisors was important. This was because if one needed to get reduced workload or off duty in case of a medical appointment, the supervisors would be considerate.

“*If you tell the in-charge, you may be favoured, but if you don't tell anybody, people will start complaining that you don't work. So there is no way your friends can favour you when the in-charge doesn't know*." FGD females

However, there was fear that supervisors might fire them or breach their confidentiality. HCWs from PNFPs were particularly against disclosing to supervisors, raising fears of losing their jobs after they were known to be positive, as was explained by one FGD participant from a PNFP facility.

‘*I think to the in-charge it would be very difficult to disclose. You never know how he/she is going to take it? Are you going to lose your job? Will there be discrimination? Are you going to be isolated? There is always that fear*.’ -FGD males

Much as HCWs reported that disclosure (to both colleagues and supervisors) was difficult, they recognized that it was beneficial in terms of offering psychological support.

“*And I think disclosing is a form of psychotherapy. So psychologically you will be relieved. You will be relieved if you share it with somebody. That person may counsel you, encourage you, and comfort you somehow. So disclosing is important only that we don't trust the people we should disclose to*." FGD males

### Preferences for accessing HIV services

Almost half of the respondents 225/499 (45%) preferred to be treated outside the facility where they worked if they were HIV infected, (Table 2). Sixty one per cent of the men preferred to be treated in their own facility compared to 53% of females, (p = 0.10). In a multivariable model, older age APR = 1.75; CI = 1.30–2.35 was positively associated with reported preference to be treated for HIV within their workplaces, (Table 4). On the contrary, being female, APR = 0.85; CI = 0.73–0.99, was associated with less preference to be treated for HIV at the workplace.

**Table 4:** Bivariate and multivarable analysis for preference to be treated for HIV at the workplace among health care workers in Wakiso and Mukono in Uganda.

Characteristics	Preference to be treated at workplace (%)	Crude PR (95% CI)	Adjusted PR (95% CI)	P-value
**Age**				
15–24	24/64 (37.5)	1	1	
25–34	90/180(50.0)	1.33(0.95–1.86)	1.31(0.94–1.83)	0.12
35–44	73/123 (59.4)	1.58(1.12–2.23)	1.59(1.12–2.24)	0.01
>45	87/132 (65.9)	1.76(1.29–2.38)	1.75(1.30–2.35)	<0.01
**Sex**				
male	85/140(60.7)	1	1	
female	189/359 (52.7)	0.85(0.73–1.04)	0.85(0.73–0.99)	0.045
**Ownership**				
Government	185/331 (55.9)	1	-	
PNFP	89/168 (53.0)	0.95(0.77–1.16)		-
**Facility level**				
Hospital	134/221(60.0)	1	1	
HC IV	50/114 (43.9)	0.72(0.56–0.94)	0.74(0.58–0.94)	0.02
HC III	90/164 (54.9)	0.91(0.71–1.16)	0.91(0.74–1.13)	0.39
**District**				
Wakiso	129/257 (50.2)	1	1	-
Mukono	145/242 (59.9)	1.19(0.93–1.15)	1.14(0.94–1.39)	0.17
**Cadre**				
Non-Clinical	46/93(49.5)	1	-	-
Clinical	228/406 (55.2)	1.14(0.87–1.47)		

CI = Confidence Interval, HC = Health Centre, PR = Prevalence Ratio.

FGD participants also reported preference to be tested for HIV outside their workplace because they feared that colleagues and patients would know their HIV status, if they were infected. The majority suggested that there should be a special clinic for HCWs, run by trustworthy people in every district. There was a preference by some respondents for HIV positive staff to work in these clinics. Some suggested an outreach arrangement where HCWs from outside their facility provide HCT, and those who test HIV positive access treatment in another facility. In order to avoid stigma, participants proposed that this clinic should also provide non HIV services.

‘*No, it shouldn't be a unit just specializing in testing or treating HIV. It should also be able to handle me, when I am pregnant. Anything, whether headache, should also be handled there. So that you confuse whoever sees you*."- FGD females

In addition, it was mentioned that health education would be important in order to stop stigmatization of HIV patients and colleagues.

### Utilisation of TB services

None of the respondents reported being routinely screened for TB infection and disease. However, five per cent (27/499) of the respondents reported a history of TB treatment in the past five years. There were no significant differences in reporting a history of TB by cadre, age, sex, type of facility or district.

### Preferences for TB services

The majority of the respondents (89%, 442/499) were willing to be screened for TB (infection and disease), with 23% (100/442) reporting a preference to be screened in a facility where no one knew them and the rest (77%) in a facility where they worked. Women were more likely to report preference for TB screening outside their place of work than men, 24% (76/314) versus 17% (22/126), respectively (p = 0.12). Results from FGDs also showed that there was very little sensitivity around TB diagnosis or treatment at the workplace. Almost all FGDs preferred that TB screening and treatment be done within the facilities where they worked. Reasons for wanting to be screened in their own facilities included: TB is not as stigmatizing as HIV and that anyone can get it through air, unlike HIV. Also being HCWs, it was felt that they were at risk of TB infection. In addition, HCWs felt that accessing TB services in their facilities would be convenient, cheap and easy.

“*With TB I think it's a bit easy; it's not so much stigmatized because those who are HIV negative also get it. There's not much stigma even in the unit where you work.*’ FGD females

“‘*It can be done [TB screening] from the workplace. Because it will be easy to access the services. Any time you can approach the person concerned and he/she will provide the service, unlike when you have to go to another facility and lineup. Travelling to another facility is also expensive, because it requires money for transport. So I think where one works, it is very easy and cheap.*’ FGD Men

The HCWs, who reported preference for TB screening outside their workplace, thought that those who would be diagnosed with TB would be suspected to have HIV and might be stigmatised. Even where screening was accepted at the workplace, majority of HCWs mentioned that TB screening and treatment should be confidential.

## Discussion

This study found that almost all HCWs had ever tested for HIV. The level of HCT utilization in our study is much higher than that reported in other similar studies conducted in Malawi, Rwanda and Zambia [10], [24], [25]. However, there were challenges faced by providers in accessing the testing services, largely related to the desire to maintain confidentiality of their HIV status. Almost half of the respondents (either tested away from their workplace or self-tested). The qualitative interviews highlighted that some of the HCWs who tested at their workplace disguised their blood specimens. Similarly, more than a third of the HCWs were not willing to disclose a positive HIV status to a supervisor and about half preferred to receive HIV care and treatment outside their workplace.

Both quantitative and qualitative findings indicate HCWs concerns about the confidentiality of their results within their own health facilities. This fear of using HIV counselling and testing services at their own workplaces has been reported in other studies [10], [26], particularly where one suspects that they could be HIV positive. Those in doubt of their status tend to test outside their workplaces [26]. These fears and preferences have implications for various interventions related to the reduction of nosocomial TB among HCWs. Relocation of HIV infected staff to safer working areas as recommended by WHO and ILO [4], is complicated if the people that make these decisions (e.g. their supervisors) are not aware of the HCWs HIV status. Similarly, HCWs who self-test or disguise their samples and do not disclose their HIV status to other HCWs may miss out on both TB and HIV services including cotrimoxazole prophylaxis, ART and IPT [27]. Our study findings show a tendency towards more reluctance to disclose HIV positive status to supervisors among HCWs from PNFP facilities, partly due to concerns about job security. This difference could be attributed to the proximity of the employer to the HCWs and the tenure of employment (easier to terminate in PNFPs than in public facilities).

Relocation of HIV infected HCWs to ‘safer areas’ in settings with high TB and HIV prevalence presents several challenges. HCWs may not want to be relocated because they may fear that people will suspect they have HIV infection and that they will have to face stigma and discrimination. Furthermore, there might not be any ‘safer areas’ in regions with high TB prevalence and weak infection control measures. In addition, in countries such as Botswana where more than one third of HCWs are HIV positive [28], [29], such a policy may require switching placements for a substantial number of HCWs. This highlights the practical challenges associated with implementing the WHO recommendation of relocating HCWs and the need to implement universal measures (administrative, ventilation and respiratory protection) to benefit all HCWs. However, such universal measures may fail to accommodate certain TB IC measures such as IPT for HIV infected HCWs, and access to other HIV related care and treatment.

Studies have shown that disclosure can be psychologically therapeutic [30], [31]. HCWs involved in our FGDs also felt that disclosing was important in offering them a psychological wellbeing. Secrecy and concealing disclosure have been reported to result in psychological distress, isolation and negative impact on an individual's daily life [30], [32], [33]. Our results show that fear of stigma was commonly reported among women and young people. This is consistent with findings elsewhere [34], [35]. Despite the benefits cited by HCWs, there was still low willingness to disclose HIV positive status which illustrates the stigma towards HIV among HCWs [4]. Additionally, in settings like Uganda and other sub-Saharan countries, where TB IC and HIV care services for HCWs are not well developed, HCWs may not see direct benefits to disclosure an HIV positive status. HCWs are bound to weigh the benefits of such disclosure against their confidentiality concerns and may choose not to disclose. Reducing HIV related stigma and discrimination in health facilities and ensuring comprehensive confidential services for HCWs should thus be of a concern.

Our findings indicate that HCWs prefer integrated occupational health services, if possible away from the workplace, in order to ensure confidentiality. The preference to access HIV care outside their workplace is contrary to the recommendation to provide such care at the workplace, which has been found to be cost-effective [36], [37]. Offering HCWs a package of services not only for HIV and TB but also for infectious and non-communicable diseases may be the way to go. This is in line with the WHO policy of integration of health services [38]

This study has some limitations. The study was only performed in 2 semi-urban districts which may not be representative of the other districts in Uganda. The proximity of these districts to the capital city (Kampala) may be associated with better access to public and private health facilities. However, this and other studies have shown that the utilization of HIV services by HCWs is limited more by confidentiality concerns than physical access [9], [27], [39]. We did not ask about the HIV status of the HCWs who had ever tested, and did not ask about disclosure and outcomes of disclosure. However, the study highlights key concerns and preferences by HCWs, which need to be addressed in the effort to scale up TB IC programs. The use of FGDs to discuss sensitive issues could also be a limitation in this study; however the participants were not selected based their HIV status. In addition, in order to get HCWS opinions on preferences for their HIV and TB services, we felt FGDs would be better. Finally, information where TB treatment was provided for those with a history of TB was not captured. However, the majority (77%) of the respondents were willing to be screened for TB at their workplace.

## Conclusions

This study highlights the need to set up integrated and comprehensive occupational health services as well as interventions for stigma reduction among HCWs in the TB IC efforts. Since women represent the majority of health care staff in most settings, workplace infection control efforts need to reflect the differential effects of HIV stigma by gender and age. In addition, universal precautions for TB IC need to be reinforced as they appear more feasible in the short term.
